# P045. OnabotulinumtoxinA: long term treatment for chronic migraine with medication overuse

**DOI:** 10.1186/1129-2377-16-S1-A183

**Published:** 2015-09-28

**Authors:** Simona Guerzoni, Maria Michela Cainazzo, Luigi Alberto Pini

**Affiliations:** Headache and Drug Abuse Inter-Department Research Centre, University of Modena e Reggio Emilia, Modena, Italy

## Introduction

Chronic migraine represents the most disabling condition among headaches, in particular when migraine is associated with drug abuse.

Patients with chronic migraine (CM) coming to our centres are difficult to treat, both because of their refractory to antimigraine prophylactic treatment and for the combination of several comorbidities, that often need a multidisciplinary approach that leads to a multi-prescription of drugs.

The treatment with OnabotulinumtoxinA (Botox^®^) is an important therapeutic option both for its efficacy in the long term, and for the safety profile, due to the lack of clinically significant side effects.

## Materials and methods

In our Headache Centre we performed a retrospective study including a sample of 67 patients with a diagnosis of CM associated with drug abuse according to the ICHD-III (beta) classification. The patients were treated with OnabotulinumtoxinA according to the paradigm of the PREEMPT study (155 U to 31 injection sites) [1].

The purpose of our study was to evaluate the duration of the Botox's efficacy in terms of headache days (HD), analgesic consumption (AC) and to assess the patients’ quality of life by some self-administered scales (SF-36, HIT-6) and pain scale (VAS) [2].

We recorded medical charts for 67 patients. However, we report the data concerning the results of only 57 patients since they represent the ones who were injected regularly every 3 months without interruption, some of them being injected up to cycle 7. Ten patients discontinued for regulatory reasons.

## Results

Positive trend of the effectiveness of the treatment appears to be significant in all parameters evaluated as shown in the table 1.

**Table 1:** Table 1

TEST	T-0	T-6	T-12	T-18
**HD**	0.98±0.09	0.77±0.30	0.69±0.29	0.65±0.36
**AC**	1.79±1.59	1.33±1.90	0.70±0.43	0.61±0.42
**HIT-6**	63.95±6.91	62.14±8.06	58.55±9.41	52.29±8.69
**SF-36 MENTAL**	48.30±21.68	51.71±22.35	59.42±21.16	73.90±20.26
**SF-36 PHYSICAL**	46.35±18.91	49.17±19.90	52.58±24.69	70.18±23.22
**VAS**	7.98±1.26	6.02±1.89	5.13±1.61	4.25±1.49

Legend:

Data are expessed as mean +/- SD. Results differ significantly vs T0. p < 0.001 (ANOVA)

HD = Headache days

AC = Analgesic Consumption

HIT-6 = Headache Impact Test

SF-36 = Short-Form Health Survey (Physical and Mental)

VAS = Visual Analogue Scale

## Conclusions

This retrospective study confirms the safety and tolerability profile of repeated treatment with OnabotulinumtoxinA and shows a good consistency of the therapeutic effect over one year of treatment. The trend of the clinical parameters suggests other studies to further investigate the long-term efficacy of the treatment, as recently suggested by Pascual [3].

Moreover, it is important to outline that in our sample we did not register any clinically relevant side effect, besides slight pain in the site of injection, and two cases of transient hypotension during the injection protocol, spontaneously reversed.

Written informed consent to publish was obtained from the patient(s).
